# A Predictive Model for Diabetic Foot Ulcer Outcome: The Wound Healing Index

**DOI:** 10.1089/wound.2015.0668

**Published:** 2016-07-01

**Authors:** Caroline E. Fife, Susan D. Horn, Randall J. Smout, Ryan S. Barrett, Brett Thomson

**Affiliations:** ^1^ U.S. Wound Registry, The Woodlands, Texas.; ^2^ Institute for Clinical Outcomes Research, Salt Lake City, Utah.; ^3^ University of Utah School of Medicine, Salt Lake City, Utah.

## Abstract

**Objective:** To develop a healing index for patients with diabetic foot ulcers (DFUs) for use in clinical practice, research analysis, and clinical trials.

**Approach:** U.S. Wound Registry data were examined retrospectively and assigned a clear outcome (healed, amputated, etc.). Significant variables were identified with bivariate analyses. A multivariable logistic regression model was created based on significant factors (*p* < 0.05) and tested on a hold-out sample of data. Out of 13,266 DFUs from the original dataset, 6,440 were eligible for analysis. The logistic regression model included 5,239 ulcers, of which 3,462 healed (66.1%). The 10% validation sample utilized 555 ulcers, of which 377 healed (67.9%).

**Results:** Variables that significantly predicted healing were as follows: wound age (duration in days), wound size, number of concurrent wounds of any etiology, evidence of bioburden/infection, patient age, Wagner grade, being nonambulatory, renal dialysis, renal transplant, peripheral vascular disease, and patient hospitalization for any reason.

**Innovation:** We present a validated stratification system, previously described as the Wound Healing Index (WHI), which predicts healing likelihood of patients with DFUs, incorporating patient- and wound-specific variables.

**Conclusion:** The DFU WHI is a comprehensive and user-friendly validated predictive model for DFU healing. It can risk stratify patients enrolled in clinical research trials, stratify patient data for quality reporting and benchmarking activities, and identify patients most likely to require costly therapy to heal.

**Figure:**
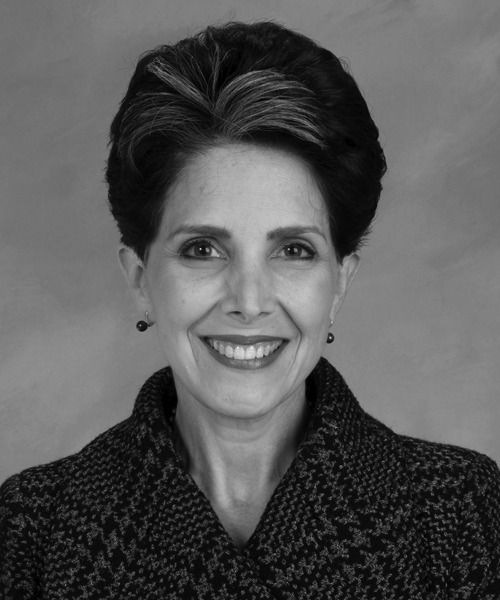
**Caroline E. Fife, MD**

## Introduction

The lack of an easy-to-use, practical, and validated method that can comprehensively risk stratify patients with diabetic foot ulcers (DFUs) has led to exclusion of patients with serious comorbid conditions from randomized controlled trials directed at the treatment of DFUs, thus limiting the generalizability of the results.^1^ There is also a clear need to include such patients in clinical trials to simulate a more real-world environment.^2^ Moreover, new reimbursement systems focused on healthcare outcomes necessitate a patient risk stratification system to adjust for differences in health status among patients, making it possible to compare provider performance fairly. The Centers for Medicare and Medicaid Services (CMS) acknowledges benefit to analyzing real-world data,^3^ and the Institute of Medicine promotes the mining of electronic health record (EHR) data for clinical research.^4^ Wound care centers have joined a national clinical data research network (CDRN),^5^ in which data are submitted to the U.S. Wound Registry (USWR), which used them to develop and validate the Wound Healing Index (WHI™),^6^ to accomplish this latter goal in wound care.

Predictive factors of DFU healing have been studied mainly with simple logistic regression models using one predictor at a time; for example, the effect of wound size (depth, area, and diameter),^7–15^ initial treatment response,^10^ or the percentage of wound area reduction at 4 weeks.^8^ Other predictive factors include the severity of the ulcer grade,^11,12,16–18^ wound duration,^12–14,19^ wound infection,^15,20–22^ elevated serum creatinine levels,^20^ previous amputation,^21^ dialysis, and peripheral arterial disease.^23–26^ Previously developed wound scoring systems combine several factors and allocate points to each factor to allow the clinician to estimate healing or amputation likelihood based on an interpretation of the total score, including the lower extremity amputation (LEA) score,^21^ the DEPA score,^22^ the MAID score,^14^ and the American Diabetes Association's DFU risk stratification.^26^ However, all of these systems have limitations. Likewise, complex multivariable mathematical models can also be utilized to predict the likelihood of DFU healing,^27–33^ but as they are theoretical in nature they are not used in patient care.^34^

## Clinical Problem Addressed

A comprehensive and practical model is needed to be used in patient care settings to identify wounds most at risk for nonhealing, to be useful in Physician Quality Reporting System (PQRS) reporting, and to classify patients most likely to require costly therapeutic interventions. At the same time, it must have further capability to be utilized as a stratification variable in clinical trials and be used as a summary variable in statistical analysis of such trials or wound care outcome datasets.

## Materials and Methods

### Settings and database description

We previously described the USWR, an aggregate national database used to create the WHI models.^6^ Data originate from a specialty-specific EHR which, at the time of analysis, met the standards for Stage 1 of “meaningful use” as defined by CMS and certified under the “HITECH” Act (Health Information Technology for Economic and Clinical Health Act) legislation by the Office of the National Coordinator for Health Information Technology.^35^ At the time of project initiation, the USWR comprised data from 56 clinics in 24 states.

This study was approved by the USWR-independent Institutional Review Board, The Woodlands IRB, which determined that this study was exempt from the requirement for patient consent because of the use of retrospective analysis of HIPAA–de-identified compliant data. This study complied with the 1975 Declaration of Helsinki.

### Identification of DFUs

Within the EHR, wounds and ulcers were defined by the ICD-9-CM code, although diabetic foot ulcer does not have a specific code. DFUs are designated in the EHR as being “chronic ulcers” that are specifically “related to” the condition of diabetes. Only foot ulcers specifically indicated by the clinician as being related to the underlying disease of diabetes were included in the DFU dataset. Physicians and nurses performed point of care electronic charting with the patient in the examination room. They also provided free text data entries, which designated the wound's specific location on the body (*e.g.,* right first metatarsal head). Thus, text field searches were used to establish right vs. left and exact ulcer location.

Additional inclusion criteria in analyses included the following: each ulcer had ≤2 clinical encounters; ≤5 days between first and last encounter; no gap between any two clinic visits was not >90 days; ≥1 wound area measurement or a clinician statement of ulcer outcome; ≥1 wound assessment with a wound area ≥t0.25 cm^2^; a date of onset for the ulcer; and the ulcer had a location on the body specified.

### Dependent variable

Previously, we published a detailed explanation of the way in which healing was defined.^6^ In those cases in which no outcome was assigned by the clinician at the final visit, longitudinal data analyses were performed to assess the change in ulcer size over time and the change in tissue type exposed over the course of care to establish which wounds had healed. Outcomes of amputation and cases of death before healing were considered not healed. Out of 13,266 DFUs from the original dataset, 6,440 were eligible for analysis. As a result of missing initial area (area at first visit before any debridement) for 646 wounds, the sample size was reduced to 5,794 for the regression analysis, of which 3,462 healed (66.1%). The development sample consisted of 90% of the dataset or 5,239 ulcers. The 10% validation sample utilized 555 ulcers, of which 377 healed (67.9%).

### Independent variables

From prior research,^6^ we identified significant predictors of healing based on the following wound and patient characteristics: wound area at first encounter; wound age at first encounter; Wagner grade; patient chronological age at first treatment; malnutrition; peripheral vascular disease; number of past or concurrent ulcers or wounds; renal transplant or failure; indications of inflammation and/or infection in the wound; and prior amputation. Additional significant factors are shown in Table 1, along with a detailed definition of all factors.

**Table 1.** *Independent variables of patient and wound characteristics significantly associated with healing predictions for diabetic foot ulcers, based on prior analyses* (6)

*Variable*	*Definition*
FirstWoundArea	Beginning wound area in cm^2^
EpiEndHospER	Caregiver encounter ending with patient sent to emergency department or hospital
PATC_Age_atFirstTreatment	Patient chronological age at first encounter
WorstArrvScoreGrp3Bed^a^	Mobility of patient at arrival; patient bed bound at arrival
WorstArrvScoreGrp2WC^a^	Mobility of patient at arrival; patient in wheelchair
WorstArrvScoreGrp1Amb^a^	Mobility of patient at arrival; patient able to ambulate
PVD2Oct12	Peripheral vascular disease was present if after scanning eight different database tables containing initial and follow-up examination information, past medical history, surgery summaries, nursing assessments, and patient's problems, the following words or word segments were found: 440.2, 440.3, popliteal, claudication, gangrene, or rest pain, or ischemia and peripheral, or ischemia and leg
NumWounds_Strt_End	Number of wounds or ulcers that started previous to or concurrent with the index wound, but exist on the patient during the time frame the index wound is being treated
InfectBioBurden2	Signs of inflammation and/or infection in the wound as indicated by the words milky, purulent, green, or malodorous describing wound exudates or the words indurated, edematous, tender to palpation, warm to touch, or erythematous describing the periwound area
CSI_Pat_RenalFailure_Transplant	Renal failure or transplant drugs were present if after scanning five different database tables containing past medical history, surgery summaries, and patient's problems, the following words or word segments were found: ESRD, CHD, CRI, end-stage renal, dialysis, hemodialysis, kidney and failure, renal and failure, or renal and transplant.
Wagner2DeepUlcer, Wagner3DeepTissue, Wagner4LocalOr5Gagrene	Wagner grade from Wagner classification at first encounter, as well as the worst during the wound episode. Each set was used in its respective model.
WoundAgeAtFirstEncounter	The number of days from wound onset to the first encounter date.

^a^ The variables are mutually exclusive and are positive for the worst condition during the wound episode (whole course model). A second set of variables was created for use in the first encounter model based on mobility at first encounter arrival.

### Data analysis

Descriptive statistics were used in the first phase of analysis to analyze categorical patient, wound, and outcome measures frequencies. We calculated the average, median, quartiles, and amount of variation (standard deviation and range) for continuous measures. Next, the relationship between each candidate predictor and the healed outcome was tested by bivariate analyses. To determine the significance of bivariate associations among discrete variables, we developed contingency tables and used chi-squared tests, Fisher's exact tests, or (for ordered categories) Wilcoxon tests to determine significance of bivariate associations. We used correlation, two-sample *t*-tests, or analysis of variance for continuous variables. We considered a two-sided *p*-value <0.05 statistically significant. Upon defining the dichotomous outcome of healed, 10% of the DFUs were randomly selected to be used for model validation. The unit of analysis used in this study is the wound.

On the remaining 90% of the DFUs, we carried out multivariable logistic regression for the dichotomous outcome of healed. In addition, with data from different time frames, we developed two healing likelihood models using the following: (1) data available at the first encounter for one model (suitcase model) or (2) data available from the whole course of care for the second model. For the development and validation samples, the time frames were identical and all patients overlapped between them.

On the basis of information available in the published literature and clinical experience, we allowed potential predictors to enter the models with stepwise selection, but only significant variables were retained. We used Spearman correlations to confirm that there were no collinear independent variables in the final models. All correlations between independent variables were <0.75. We measured discrimination of the two models (the first visit and the all visits development models using the 90% sample) using the area under the receiver operator characteristics curve (c statistic) to analyze the ability of the model in distinguishing DFUs that did not heal from DFUs that did heal.

The DFU WHI provides the predicted healing probability of a specified DFU (without regard to any time constraint), which is based on the multiplication of the logistic regression parameter estimates with the values of the significant DFU variables. We used the 10% validation sample to validate the WHI. In the 10% validation model, we also used the Hosmer–Lemeshow goodness-of-fit test to determine the degree of correspondence between probabilities of achieving the outcome (healed) estimated by the WHI and the actual outcome proportion over groups spanning the entire range of probabilities (calibration). The USWR team directed the Institute for Clinical Outcomes Research (ICOR) team on their performance of the analyses, which were done with SAS version 9.2 (SAS Institute, Inc., Cary, NC).

In addition, all eligible DFUs used in the analysis (*n* = 5,794) were divided into two sets by number of wounds treated by an individual physician, using 30 as the cut point. This enabled us to examine complete wound healing according to the WHI score (breakpoints ≤33, >33–67, and >67).

## Results

There were 13,226 DFUs in the original dataset spanning a time frame from July 2003 to July 2011. In addition to those ulcers not meeting the inclusion criteria, some additional ulcers were excluded because the clinicians determined that the patient was lost to follow up, leaving 6,440 DFUs for analysis (48.7% of the original DFU dataset) (Table 2).

**Table 2.** Data cleaning steps

*Step*	*Cleaning Step*	*Diabetic Foot Ulcers*
1	Starting number of ulcers/wounds	13,226
2	Wound location not specified adequately for analysis	−1,231
3	No encounter data	−125
4	Delete when encounter date is after resolved date	−0
5	Require more than one wound encounter	−1,553
6	Require that first encounter date is not resolved date	−0
7	Keep wounds where longest gap between encounters is <90 days	−936
8	Require days between first and last encounter ≥5	−211
9	Wound outcome group “Throw out” (lost to follow-up)	−238
10	Require wound age	−0
11	No areas, no evidence of outcome	−71
12	Evidence status = none and MeasureStat2 = depth or no	−1,423
13	Max wound area <0.25 cm^2^	−998
14	Encounter date duplicates with nonidentical data—keep worst	−0
15	Encounters after resolved date	−0
	End number of ulcers/wounds	6,440

Table 3 shows all the variables that were examined to assess their bivariate association with a DFU being healed for the 6,440 DFUs that were eligible for analysis. Many were significantly associated with DFU healing likelihood. Table 3 also shows which bivariate variables were significant in the final development regression model of DFU likelihood of being healed.

**Table 3.** *Bivariate analyses of all variables studied in diabetic foot ulcer models (*n* = 6,440) with sign in parentheses indicating the direction of the bivariate association and bivariate significance probability for each predictor variable with outcome of healed*

*Variable*	*Significant in Final DFU Regression Models*	p*-Value*
Infection/bioburden	Yes	(−)<0.001
Patient admitted for acute hospital stay or emergency department visit	Yes	(−)<0.001
First wound area (healed wound associated with smaller area)	Yes	(−)<0.001
Patient age at first treatment (healed wound associated with younger age)	Yes	(−)<0.001
Renal transplant or dialysis	Yes	(−)<0.001
Wagner grades^a^	Yes	<0.001
Number of previous or concurrent other wounds or ulcers (healed wound associated with fewer other wounds)	Yes	(−)<0.001
Mobility of patients at arrival—bed bound vs. wheelchair or able to ambulate	Yes	(−)<0.001
Peripheral vascular disease	Yes	(−)<0.001
Wound age at first encounter	Yes	(−)<0.001
Patient is on dialysis	No	(−)<0.001
Insulin-dependent diabetes	No	(+)0.979
Patient takes pain medications	No	(−)0.377
Paralyzed	No	(−)0.448
Renal transplant	No	(−)0.382
Wound location^a^	No	<0.001
Days from first to last encounter (+: healed wound associated with longer time)	No	(+)<0.001
Worst Braden score (+: healed wounds associated with higher score)	No	(+)<0.001
Malnutrition	No	(−)0.001
Braden malnutrition subset (+: healed wounds associated with higher score)	No	(+)<0.001
Autoimmune disease	No	(+)0.014
Patient on muscle relaxants	No	(+)0.005
Prior amputation	No	(−)<0.001
Patient resides in a nursing home or skilled nursing facility	No	(−)0.001
Dementia and Alzheimer's	No	(−)0.002
Autoimmune disease and rheumatoid arthritis	No	(+)0.024
Incontinence	No	(−)<0.001
Worst Braden subscore for mobility (+: healed wounds associated with higher score)	No	(+)<0.001
Number of foot pulses obtained by Doppler rather than being palpable (+: healing associated with higher number)	No	(+)0.018
Patient is male	No	(−)0.265
Patient takes transplant anti-rejection drugs	No	(+)0.884
Any organ transplant	No	(−)0.528
Alcoholic liver disease	No	(+)0.496
Current smoker	No	(−)0.135
Sleep apnea	No	(+)0.872
Wound on left side	No	(−)0.559
BMI category of patient at first treatment^a^	No	<0.001

^a^ No direction of association provided since this variable has multiple categories.

BMI, body mass index; DFU, diabetic foot ulcer.

The suitcase model (the patient and wound factors present at initial assessment) was created using 90% of the data (5,794 ulcers) and retaining 10% of data for model validation. The variables that significantly predict likelihood of being healed for DFU in multivariable logistic regressions are presented in Table 4. All regression coefficients were negative—meaning that they were associated with less likelihood of being healed. The variables in Table 4 are ordered from the strongest significant predictor to the weakest significant predictor for each model: whole course and first encounter.

**Table 4.** Multivariable logistic regression model and fit statistics to predict healed (yes/no) for 90% development sample for diabetic foot ulcers

*Number of Wounds = 5,239 Number Healed (%) = 3,462 (66.1%)*	*Estimate Direction*	*Wald Order*^a^	p*-Value*	*c Statistic*^b^
Whole course model				0.668
Wagner grades 4 or 5 (local or extensive gangrene)	—	1	<0.0001	
Wagner grade 3 (deep tissue)	—	2	<0.0001	
Wound age at first encounter	—	3	<0.0001	
Wagner grade 2 (deep ulcer)	—	4	<0.0001	
Renal transplant or dialysis	—	5	<0.0001	
First wound area	—	6	<0.0001	
Patient age at first treatment	—	7	<0.0001	
Infection/bioburden	—	8	<0.0001	
Mobility of patients at arrival—wheelchair	—	9	<0.0001	
Number of previous or concurrent other wounds or ulcers	—	10	0.0003	
Mobility of patients at arrival—bed bound	—	11	0.0364	
Patient admitted for acute hospital stay or emergency department visit	—	12	0.0443	
Peripheral vascular disease	—	13	0.0840	
First encounter model				0.648
Wagner grades 4 or 5 (local or extensive gangrene)	—	1	<0.0001	
Wound age at first encounter	—	2	<0.0001	
First wound area	—	3	<0.0001	
Renal transplant or dialysis	—	4	<0.0001	
Wagner grade 2 (deep ulcer)	—	5	<0.0001	
Wagner grade 3 (deep tissue)	—	6	<0.0001	
Patient age at first treatment	—	7	<0.0001	
Mobility of patients at arrival of first visit—wheelchair	—	8	<0.0001	
Peripheral vascular disease	—	9	0.007	
Mobility of patients at arrival of first visit—bed bound	—	10	0.011	

^a^ Most significant = 1 to least significant.

^b^ Performance metric of model discrimination equivalent to the area under the receiver operating characteristic curve.

Table 5 shows the performance of each DFU model in the validation dataset. Both the “whole course of care” and “first encounter” models validated well. Both c statistics were >0.65 and we did not find that the Hosmer–Lemeshow test was significant, indicating that both the DFU first encounter and all visit models fit the data in the independent validation sample very well. Table 6 lists the 10 questions that are used to produce the WHI for diabetic ulcers.

**Table 5.** Logistic regression model and fit statistics of the Wound Healing Index to predict healed (yes/no) for 10% validation sample for diabetic foot ulcers

*Number of Wounds = 555* *Number Healed (%) = 377 (67.9%)*	*Estimate Direction*	p*-Value*	*c Statistic*^a^	*Hosmer–Lemeshow* p*-Value*
Whole course model			0.662	0.489
Wound Healing Index	+	<0.0001		
First encounter model			0.659	0.157
Wound Healing Index	+	<0.0001		

^a^ Performance metric of model discrimination equivalent to the area under the receiver operating characteristic curve.

**Table 6.** Questions to produce diabetic foot ulcer Wound Healing Index (see Table 1 for more details)

*Number*	*Question*
1	Patient age in years (calculated from date of birth) at first treatment
2	Wound age (duration) in days (calculated from wound onset) at first encounter
3	Wound area in cm^2^ (calculated from length × width) at first encounter
4	What is the patient's primary ambulatory method? (walks unaided, cane, crutches, walker, roll about, scooter, wheelchair bound, bed bound)
5	Was the patient admitted to the hospital or the emergency department on the date of service?
6	How many total wounds or ulcers of any type does the patient have?
7	Does this wound have evidence of infection or bioburden? (evidenced by purulent, green, malodorous drainage, periwound induration, tenderness to palpation, warmth)
8	Is the patient on dialysis or status postrenal transplant?
9	What is the Wagner grade of the ulcer (1–5)?
10	Does the patient have peripheral vascular disease (claudication, rest pain, abnormal arterial vascular studies, loss of pulses)?

When DFUs were divided by the number of wounds treated by individual physicians, for physicians who had treated ≤30 wounds (*n* = 1,325), the percentages of wounds healed according to the WHI categories (≤33, >33–67, >67) were 35.3%, 50.1%, and 72.8%, respectively. The results for the first WHI category may have considerable imprecision as only 17 wounds were in the first category for the group of physicians treating ≤30 wounds. In contrast, for physicians treating 31 wounds or more, the corresponding figures were 30.2%, 57.2%, and 78.0% for the same WHI categories.

## Discussion

The development of a composite score or parameter to predict healing chronic wounds has occupied the best minds in wound care research for many decades.^15,19,21,22,26,36,37^ Previously, the most comprehensive study to validate a risk score was carried out by Lipsky *et al.*^21^ on 3,018 patients hospitalized for diabetic foot infection, with a greater likelihood of having an LEA. There were 11 significant risk factors identified, among which the strongest predictors were the presence of infection and peripheral vascular disease. A simple LEA risk score was developed around five parameters that strongly correlated with LEA rates of 0% for patients of score 0 and ∼50% for those of score ≥21. However, the Lipsky study suffered from a potential selection bias and the inability to capture other potentially significant factors from the records, such as a history of previous lower extremity revascularization procedures. In one of the largest DFU cohort analyses to date (31,000 patients), Margolis *et al.* concluded from multivariate logistic regression that the initial wound size, wound duration, and Curative Health Services ulcer grade were predictors of failure to heal,^11^ but no composite score or risk algorithm was included in the publication of the results.

The DEPA score has also been validated in literature in terms of healing likelihood and risk of LEA.^20^ Of the 84 patients included in the study, those with DEPA scores ≤6 had excellent healing, and a score ≥10 indicated poorer healing rates (in 85% of the patients). The MAID score^19^ evaluates for the presence of multiple ulcerations (M), wound area (A), palpable pedal pulses (I), and ulcer duration (D). In this study, 2,019 patients with 4,004 wounds were divided into subgroups with the same score to validate the tool. Each one-point score increase reduces the chance for healing by 37%, but there were not as many variables taken into consideration as in our present study. Using the four data elements included in the MAID index, the c statistic was 0.60 for our DFUs compared to 0.67 for the DFU WHI.

The WHI has been embedded within a wound care-specific EHR allowing clinicians to identify patients with wounds that are unlikely to heal spontaneously so they can be prioritized for advanced therapeutics. It is already being used to stratify wounds into risk categories for reporting wound outcome data as part of the PQRS. It is also being used to create matched cohorts for both prospective and retrospective clinical research to enable real-world studies of product effectiveness. In addition, it could be used by third party payers to identify patients who are most likely to require additional healthcare resources to achieve positive outcomes.

The WHI has practical utility because it not only considers the parameters incorporated in other wound scoring systems, such as wound size, severity, duration, depth, but also numerous patient factors, some of which have not been previously identified as being significant predictors of outcome, including the usual method of transportation, which may be a surrogate for debility. This inclusive model was made possible by the fact that the entire EHR of all patients from all participating clinics was transmitted to the registry (*e.g.,* patient social history, patient medical history, patient surgical history, functional assessments, nutritional assessments, physical examination, medications, wound history, and interval hospitalizations) in a format structured to facilitate subsequent data analysis. On the clinical side, data capture occurred in a uniform manner since all clinics used the same EHR and, importantly, both advanced practitioners and nurses performed point of care charting (with the patient in the examination room) using an EHR that also internally calculated billed charges. As a result, numerous potential factors could be systematically explored. An advantage to the CDRN is that the registry includes the participation of every patient seen at each clinic. Therefore, patient enrollment has no selection bias. Another advantage of the CDRN is that it prevents the artificial inflation of patient outcomes to improve the clinic's reported “healing rate” because *post hoc* vetting of outcome information using these data is avoided, since the data are the actual medical record of each patient. We designed this study to identify inherent patient and wound characteristics that are associated with the likelihood of healing. This study was not meant to assess treatment impact; thus, we did not need to control for variations in care, which undoubtedly existed among the clinics.

There are significant limitations to this project. Only 48.7% of the original DFU dataset (*n* = 6,440) was analyzed in this study. The quality and consistency of clinical input into the EHR may affect the data. However, there is an incentive for charting completeness (without regard to motivation for research on part of the physician or the facility), because the EHR internally audits the chart to determine both the facility and the physician level of service. Unfortunately, important ancillary information likely related to healing outcome such as HbA1C was not consistently available. It is hoped that the progressive governmental requirements of “meaningful use” of certified EHRs (currently at stage 2) will expand the data available to the CDRN as clinicians and hospitals are incentivized to create interfaces to store electronic healthcare information. Finally, although the USWR data are national, this does not automatically generalize results to the U.S. population despite the fact that studies published using data from the USWR tend to agree with results in the literature.

These WHI predictive models are anticipated to be used in diverse ways, which is why we created two models. The first model may be used in clinical practice on the initial visit to identify hard-to-heal DFUs, perhaps to prioritize those most in need of advanced therapeutics. Or, in prospective trials, researchers could use this model for patient stratification to appropriately allocate enrolled patients to study and control groups. Models are more challenging to utilize in clinical practice than simple scoring tools since they involve more complex calculations. Our answer is to provide access to the model using the USWR website (www.uswoundregistry.com/whi). Clinicians and researchers can access the predictive model by inputting the answers to the questions shown in Table 6. The initial visit WHI is now part of the EHR associated with the CDRN, and in the future, clinicians will have access to its predictions upon the completion of a DFU patient's first encounter. The second slightly more predictive model can be used in retrospective data analysis as part of comparative effectiveness research.

## Innovation

Previous risk score algorithms published in the literature have significant limitations. The DFU version of the WHI is based on a reasonable sample size, validated, and its creation involved studying wide arrays of relevant variables. It can be used in clinical practice to identify patients who are most likely to require advanced therapeutics, as a means of risk stratification in clinical trials, as a factor in modeling of wound care research, and to risk stratify patients whose outcomes are now being reported under the PQRS.

Key Findings

• The DFU version of the WHI can be used as a validated stratification system in clinical trials, a score for use in clinical practice that reflects patient comorbidities and wound severity, or as a covariate in wound care research
• The DFU WHI predicts the healing likelihood of a given DFU
• Variables that significantly predicted healing included wound age (duration in days), wound size, number of concurrent wounds of any etiology, evidence of bioburden/infection, patient age, Wagner grade, being nonambulatory, renal dialysis, renal transplant, peripheral vascular disease, and patient hospitalization for any reason
• The DFU WHI score can be calculated using a portal on the USWR web page and will be used as a risk stratification method for PQRS reporting.

## About the Authors

**Caroline E. Fife, MD,** did her residency in Family Medicine at the University of Texas, Southwestern in Dallas, followed by a 2-year Fellowship in Undersea and Hyperbaric Medicine at Duke University, where she was subspecialty board certified in UHM by Preventive Medicine. In 1990, she joined the University of Texas Health Science Center faculty in Houston, where she was a Professor of Medicine in the Division of Cardiology until 2013, and she initiated the Memorial Hermann Center for Wound Healing and Hyperbaric Medicine. She is now the Medical Director of the St. Luke's Wound Care Clinic in The Woodlands, Texas, and a Professor of Geriatrics at Baylor College of Medicine in Houston. Since 1998, Dr. Fife has been a Certified Wound Specialist. She has participated on the boards of the Alliance of Wound Care Stakeholders, American Academy of Wound Management, the Association for the Advancement of Wound Care, the American Professional Wound Care Association, and the Undersea and Hyperbaric Medical Society. Dr. Fife is also the Chief Medical Officer of Intellicure, a Texas-based software company, and is the Executive Director of the USWR, a nonprofit company recognized by CMS as a Qualified Clinical Data Registry for PQRS reporting and measures development and which provides data for comparative effectiveness studies in wound care. **Susan D. Horn, PhD,** was an employee and stockholder of International Severity Information Systems and the ICOR in Salt Lake City, UT, when this research was conducted. She is now a Professor at the University of Utah School of Medicine. **Randall J. Smout, MS,** was an employee of International Severity Information Systems and the ICOR in Salt Lake City, UT, when this research was conducted. **Ryan S. Barrett, MStat,** was an employee of International Severity Information Systems and the ICOR in Salt Lake City, UT, when this research was conducted. **Brett Thomson, BS,** is the Chief Information Officer of Intellicure and a Director of the Chronic Disease Registry.

## Abbreviations and Acronyms

CDRN: clinical data research network
CMS: Centers for Medicare and Medicaid Services
DFU: diabetic foot ulcer
EHR: electronic health record
ICOR: Institute for Clinical Outcomes Research
LEA: lower extremity amputation
PQRS: Physician Quality Reporting System
USWR: U.S. Wound Registry
UT: University of Texas
WHI™: Wound Healing Index
